# Supplementary material for: Radiolarian assemblages in the shelf area of the East China Sea and Yellow Sea and their ecological indication of the Kuroshio Current derivative branches
Source: PeerJ. 2020 Sep 18;8:e9976. doi: 10.7717/peerj.9976 (PMC7505074; doi:10.7717/peerj.9976)
Supplement: Supplemental Information 1 [file peerj-08-9976-s003.pdf]

Software: Primer 6.0

Type of analysis: Hierarchical cluster analysis

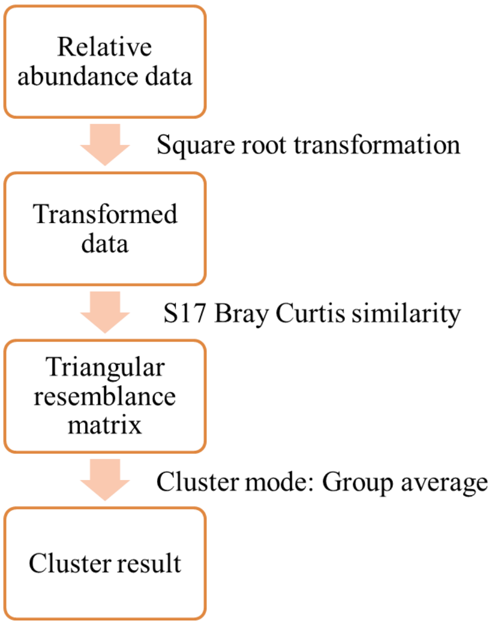

Reply figure 1. Diagram of cluster analysis procedures.

Software: Canoco 4.5

Type of analysis: DCA

Square root-transformation of species data.

```
[Wed Jul 08 13:45:15 2020] Log file created
[Wed Jul 08 13:45:38 2020] Settings change cancelled
[Wed Jul 08 13:46:49 2020] Settings changed
[Wed Jul 08 13:46:57 2020] CON file [D:\Canoco\spe.con] saved
[Wed Jul 08 13:46:58 2020] Running CANOCO:
[Wed Jul 08 13:46:58 2020] CON file [D:\Canoco\spe.con] saved
 Program CANOCO Version 4.5 February 2002 - written by Cajo J.F. Ter Braak
 (C) 1988-2002 Biometris - quantitative methods in the life and earth sciences
 Plant Research International, Wageningen University and Research Centre
 Box 100, 6700 AC Wageningen, the Netherlands
 CANOCO performs (partial) (detrended) (canonical) correspondence analysis,
 principal components analysis and redundancy analysis.
 CANOCO is an extension of Cornell Ecology program DECORANA (Hill,1979)

 *** Type of analysis ***
   Model                 Gradient analysis
                   indirect      direct      hybrid
 linear            1=PCA         2= RDA        3
 unimodal           4= CA         5= CCA        6
    ,,              7=DCA         8=DCCA        9
                   10=non-standard analysis
 Type analysis number
 Answer =    7


 *** Data files ***
 Species data          : D:\Canoco\spe.dta
 Covariable data       :
 Environmental data :
 Initialization file:

 Number of segments                    =     26
 Nonlinear recaling of axes
 Rescaling threshold                 = 0.00
 Number of axes in biplot           =       2

 Diagnostics                           =       2
```

File : D:\Canoco\spe.dta
Title : WCanoImp produced data file
Format : (I5,1X,5F14.9,27(/6X,(5F14.9)))
No. of couplets of species number and abundance per line : 0

No samples omitted
Number of samples 23
Number of species 137
Number of occurrences 797

Square root-transformation of species data
No species-weights specified
No sample-weights specified
No downweighting of rare species

No. of active samples: 23
No. of passive samples: 0
No. of active species: 137

Total inertia in species data=
Sum of all eigenvalues of CA = 1.60531

**** Summary ****

Axes		1	2	3	4	Total inertia
Eigenvalues	:	0.229	0.129	0.070	0.041	1.605
**Lengths of gradient**	**:**	**1.773**	1.421	1.164	1.052	
Cumulative percentage variance of species data	:	14.2	22.3	26.7	29.2	
Sum of all eigenvalues						1.605

Note: The gradient length of the first DCA axis was 1.773.

Type of analysis: RDA.

a. The relative abundance data was square root transformed to reduce the effect of extremely high values.

b. Thirteen environmental variables, i.e. depth, SST, SSS, oxygen, phosphate, nitrate, silicate, chlorophyll-a, particulate organic carbon, clay percentage, silt percentage, sand percentage, and mean grain size.

c. Automatic forward selection with Monte Carlo tests (999 permutations)

d. “5” was set as the maximum acceptable level of VIF.

[Wed Jul 08 13:46:58 2020] CANOCO call succeeded
[Wed Jul 08 13:48:04 2020] Settings changed
[Wed Jul 08 13:48:05 2020] Running CANOCO:
[Wed Jul 08 13:48:05 2020] CON file [D:\Canoco\spe.con] saved
Program CANOCO Version 4.5 February 2002 - written by Cajo J.F. Ter Braak
(C) 1988-2002 Biometris - quantitative methods in the life and earth sciences
Plant Research International, Wageningen University and Research Centre
Box 100, 6700 AC Wageningen, the Netherlands
CANOCO performs (partial) (detrended) (canonical) correspondence analysis,
principal components analysis and redundancy analysis.
CANOCO is an extension of Cornell Ecology program DECORANA (Hill,1979)

*** Type of analysis ***

Model	Gradient analysis		
	indirect	direct	hybrid
linear	1=PCA	2= RDA	3
unimodal	4= CA	5= CCA	6
,,	7=DCA	8=DCCA	9
	10=non-standard analysis		

Type analysis number
Answer =   2

*** Data files ***
Species data   : D:\Canoco\spe.dta
Covariable data   :
Environmental data : D:\Canoco\env.dta
Initialization file:

Forward selection of envi. variables =   1
Scaling of ordination scores   =   2
Diagnostics   =   1

File   : D:\Canoco\spe.dta
Title   : WCanoImp produced data file

Format : (I5,1X,5F14.9,27(/6X,(5F14.9)))
No. of couplets of species number and abundance per line : 0

No samples omitted
Number of samples 23
Number of species 137
Number of occurrences 797

File : D:\Canoco\env.dta
Title : WCanoImp produced data file
Format : (I5,1X,4F15.9,3(/6X,(4F15.9)))
No. of environmental variables : 13

No interaction terms defined

Squareroot-transformation of species data
No species-weights specified
No sample-weights specified
Centering/standardization by species = 1
Centering/standardization by samples = 0

No. of active samples: 23
No. of passive samples: 0
No. of active species: 137

Total sum of squares in species data = 366.587
Total standard deviation in species data TAU = 0.341086
****** Collinearity detected when fitting variable 12 ******

****** Check on influence in covariable/environment data ******
The following sample(s) have extreme values

Sample	Environmental variable	Influence	Covariable influence	+ Environment space influence
17	7	5.2x		
18	1	8.7x		
18	3	42.6x		
18	6	5.2x		
18	8	25.7x		
18	9	8.5x		

22 5 6.5x
****** End of check ******

**** Start of forward selection of variables ****

*** Unrestricted permutation ***

Seeds: 23239 945

N	Name	Extra fit
12	Clay	0.04
6	Phosphate	0.08
5	Nitrate	0.09
13	Mz	0.09
1	Depth	0.09
9	POC	0.09
4	Silicate	0.10
10	Sand	0.10
8	Chlor_a	0.11
7	Oxygen	0.11
11	Silt	0.12
3	SSS	0.13
2	SST	0.14

Environmental variable 2 tested
Number of permutations= 999

P-value 0.0040 (variable 2; F-ratio= 3.34; number of permutations= 999)

Environmental variable 2 added to model
Variance explained by the variables selected: 0.14
" " " all variables : 0.72

N	Name	Extra fit
7	Oxygen	0.04
12	Clay	0.05
13	Mz	0.09
4	Silicate	0.09
6	Phosphate	0.10

10 Sand 0.10
11 Silt 0.12
5 Nitrate 0.13
9 POC 0.16
1 Depth 0.18
8 Chlor_a 0.21
3 SSS 0.25
Environmental variable 3 tested
Number of permutations= 999

P-value 0.0010 (variable 3; F-ratio= 8.01; number of permutations= 999)

Environmental variable 3 added to model
Variance explained by the variables selected: 0.38
" " " all variables : 0.72

N Name Extra fit

6 Phosphate 0.03
8 Chlor_a 0.04
1 Depth 0.04
9 POC 0.04
12 Clay 0.04
7 Oxygen 0.04
5 Nitrate 0.04
4 Silicate 0.05
13 Mz 0.05
10 Sand 0.05
11 Silt 0.06
Environmental variable 11 tested
Number of permutations= 999

P-value 0.0170 (variable 11; F-ratio= 1.90; number of permutations= 999)

Environmental variable 11 added to model
Variance explained by the variables selected: 0.44
" " " all variables : 0.72

N Name Extra fit

1 Depth 0.03
6 Phosphate 0.03

8 Chlor_a 0.04
13 Mz 0.04
9 POC 0.04
7 Oxygen 0.04
12 Clay 0.04
10 Sand 0.04
5 Nitrate 0.04
4 Silicate 0.04

Environmental variable 4 tested
Number of permutations= 999

P-value 0.0720 (variable 4; F-ratio= 1.55; number of permutations= 999)

Environmental variable 4 added to model
Variance explained by the variables selected: 0.48
" " " all variables : 0.72

N Name Extra fit

1 Depth 0.03
8 Chlor_a 0.03
6 Phosphate 0.03
9 POC 0.03
13 Mz 0.04
7 Oxygen 0.04
12 Clay 0.04
10 Sand 0.04
5 Nitrate 0.04

Environmental variable 5 tested
Number of permutations= 999

P-value 0.0810 (variable 5; F-ratio= 1.47; number of permutations= 999)

Environmental variable 5 added to model
Variance explained by the variables selected: 0.53
" " " all variables : 0.72

N Name Extra fit

6 Phosphate 0.03
1 Depth 0.03
9 POC 0.03

8 Chlor_a 0.03
12 Clay 0.04
10 Sand 0.04
13 Mz 0.04
7 Oxygen 0.04
Environmental variable 7 tested
Number of permutations= 999

P-value 0.0610 (variable 7; F-ratio= 1.63; number of permutations= 999)

Environmental variable 7 added to model
Variance explained by the variables selected: 0.57
" " " all variables : 0.72

N Name Extra fit

13 Mz 0.02
12 Clay 0.02
10 Sand 0.02
8 Chlor_a 0.03
1 Depth 0.03
6 Phosphate 0.03
9 POC 0.03
Environmental variable 9 tested
Number of permutations= 999

P-value 0.3200 (variable 9; F-ratio= 1.13; number of permutations= 999)

Environmental variable 9 added to model
Variance explained by the variables selected: 0.60
" " " all variables : 0.72

N Name Extra fit

8 Chlor_a 0.02
13 Mz 0.02
12 Clay 0.02
10 Sand 0.02
1 Depth 0.03
6 Phosphate 0.03
Environmental variable 6 tested
Number of permutations= 999

P-value 0.2890 (variable 6; F-ratio= 1.17; number of permutations= 999)

Environmental variable 6 added to model
Variance explained by the variables selected: 0.63
" " " all variables : 0.72

N	Name	Extra fit
8	Chlor_a	0.02
13	Mz	0.02
12	Clay	0.02
10	Sand	0.02
1	Depth	0.03

Environmental variable 1 tested
Number of permutations= 999

P-value 0.4300 (variable 1; F-ratio= 1.02; number of permutations= 999)

Environmental variable 1 added to model
Variance explained by the variables selected: 0.66
" " " all variables : 0.72

N	Name	Extra fit
13	Mz	0.02
12	Clay	0.02
10	Sand	0.02
8	Chlor_a	0.02

Environmental variable 8 tested
Number of permutations= 999

P-value 0.7030 (variable 8; F-ratio= 0.78; number of permutations= 999)

Environmental variable 8 added to model
Variance explained by the variables selected: 0.68
" " " all variables : 0.72

N	Name	Extra fit
13	Mz	0.02

12 Clay 0.02
10 Sand 0.02
Environmental variable 10 tested
Number of permutations= 999

P-value 0.7170 (variable 10; F-ratio= 0.78; number of permutations= 999)

Environmental variable 10 added to model
Variance explained by the variables selected: 0.70
" " " all variables : 0.72

N Name Extra fit

13 Mz 0.02
Environmental variable 13 tested
Number of permutations= 999

P-value 0.7300 (variable 13; F-ratio= 0.72; number of permutations= 999)

Environmental variable 13 added to model
Variance explained by the variables selected: 0.72
" " " all variables : 0.72

No more variables to improve fit
*** End of selection ***

****** Collinearity detected when fitting variable 12 ******
1
**** Correlation matrix ****

SPEC AX1 1.0000
SPEC AX2 -0.0101 1.0000
SPEC AX3 0.0124 -0.0474 1.0000
SPEC AX4 0.0732 -0.0410 0.0955 1.0000
ENVI AX1 0.9821 0.0000 0.0000 0.0000 1.0000
ENVI AX2 0.0000 0.9793 0.0000 0.0000 0.0000
1.0000
ENVI AX3 0.0000 0.0000 0.9484 0.0000 0.0000
0.0000 1.0000
ENVI AX4 0.0000 0.0000 0.0000 0.8575 0.0000
0.0000 0.0000 1.0000
Depth -0.0887 -0.8537 -0.1676 -0.0567 -0.0903

						-0.8717	-0.1767	-0.0662
SST	0.4222	-0.8165	-0.1715	0.0643	0.4299	-0.8338	-0.1808	0.0750
SSS	-0.3478	-0.8215	-0.3476	0.1235	-0.3541	-0.8389	-0.3666	0.1441
Silicate	-0.1110	0.8801	0.0336	-0.2354	-0.1130	0.8987	0.0354	-0.2745
Nitrate	0.0873	0.8239	-0.0412	-0.3053	0.0888	0.8414	-0.0435	-0.3560
Phosphate	0.0713	0.7663	0.1034	-0.2034	0.0726	0.7825	0.1091	-0.2372
Oxygen	-0.3785	0.6960	0.2585	-0.2860	-0.3853	0.7107	0.2726	-0.3335
Chlor_a	0.2790	0.8163	0.3013	-0.2324	0.2841	0.8336	0.3178	-0.2711
POC	0.0110	0.8762	0.1581	-0.2337	0.0112	0.8947	0.1667	-0.2725
Sand	-0.4565	-0.2069	0.3835	0.0495	-0.4648	-0.2113	0.4044	0.0577
Silt	0.5241	0.2363	-0.3745	0.0162	0.5336	0.2413	-0.3949	0.0189
Clay	0.1920	0.0907	-0.3650	-0.2467	0.1954	0.0926	-0.3848	-0.2877
Mz	0.4262	0.1746	-0.3955	-0.0973	0.4339	0.1783	-0.4171	-0.1135

	SPEC AX1	SPEC AX2	SPEC AX3	SPEC AX4	ENVI AX1	ENVI AX2	ENVI AX3	ENVI AX4
Depth	1.0000							
SST	0.7854	1.0000						
SSS	0.8538	0.6477	1.0000					
Silicate	-0.7994	-0.8142	-0.7754	1.0000				
Nitrate	-0.7812	-0.7080	-0.7992	0.9054	1.0000			
Phosphate	-0.7097	-0.6309	-0.7712	0.9348	0.8659	1.0000		
Oxygen	-0.7246	-0.9362	-0.6337	0.7772	0.7353	0.6005	1.0000	
Chlor_a	-0.8738	-0.6624	-0.9704	0.8327	0.8544	0.8103	0.6823	1.0000
POC	-0.9140	-0.8240	-0.8815	0.9044	0.8902	0.7965	0.8132	0.9409
Sand	0.2742	0.0269	0.2320	-0.1830	-0.1591	-0.2059	0.0303	-0.2148

Silt	-0.2993	-0.0087	-0.2719	0.1837	0.1655	0.2214	-0.0674	0.2370
Clay	-0.1630	-0.0801	-0.0802	0.1586	0.1198	0.1330	0.0884	0.1198
Mz	-0.2450	-0.0241	-0.1985	0.1696	0.1586	0.1883	-0.0120	0.1909
	Depth	SST	SSS	Silicate	Nitrate	Phosphate	Oxygen	Chlor_a

POC	1.0000				
Sand	-0.1899	1.0000			
Silt	0.1929	-0.9907	1.0000		
Clay	0.1575	-0.9074	0.8419	1.0000	
Mz	0.1732	-0.9954	0.9754	0.9366	1.0000
	POC	Sand	Silt	Clay	Mz

N	name	(weighted) mean	stand. dev.	**inflation factor**
1	SPEC AX1	0.0000	1.0182	
2	SPEC AX2	0.0000	1.0212	
3	SPEC AX3	0.0000	1.0545	
4	SPEC AX4	0.0000	1.1662	
5	ENVI AX1	0.0000	1.0000	
6	ENVI AX2	0.0000	1.0000	
7	ENVI AX3	0.0000	1.0000	
8	ENVI AX4	0.0000	1.0000	
1	Depth	74.7565	16.8072	14.5845
2	SST	21.1497	1.3663	58.0160
3	SSS	33.1070	1.5214	123.1374
4	Silicate	7.0615	1.8636	61.2506
5	Nitrate	2.0589	0.8470	30.4940
6	Phosphate	0.2368	0.0477	23.7822
7	Oxygen	5.2959	0.2116	27.0383
8	Chlor_a	1.2082	1.1019	286.7826
9	POC	168.3448	59.7897	130.6158
10	Sand	0.4770	0.2480	**4057.4644**
11	Silt	0.3901	0.1932	607.5501
12	Clay	0.1329	0.0624	0.0000
13	Mz	4.4115	1.0372	1839.1205

Note: “Sand” was removed from RDA model.

**** Summary ****

Axes 1 2 3 4
Total variance

Eigenvalues : 0.321 0.106 0.071 0.049
1.000
Species-environment correlations : 0.982 0.979 0.948 0.858
Cumulative percentage variance
of species data : 32.1 42.7 49.8 54.7
of species-environment relation: 44.7 59.4 69.2 76.0

Sum of all eigenvalues
1.000
Sum of all canonical eigenvalues
0.719

[Wed Jul 08 13:48:10 2020] CANOCO call succeeded
[Wed Jul 08 13:49:31 2020] Settings changed
[Wed Jul 08 13:49:32 2020] Running CANOCO:
[Wed Jul 08 13:49:32 2020] CON file [D:\Canoco\spe.con] saved
Program CANOCO Version 4.5 February 2002 - written by Cajo J.F. Ter Braak
(C) 1988-2002 Biometris - quantitative methods in the life and earth sciences
Plant Research International, Wageningen University and Research Centre
Box 100, 6700 AC Wageningen, the Netherlands
CANOCO performs (partial) (detrended) (canonical) correspondence analysis,
principal components analysis and redundancy analysis.
CANOCO is an extension of Cornell Ecology program DECORANA (Hill,1979)

For explanation of the input/output see the manual or
Ter Braak, C.J.F. (1995) Ordination. Chapter 5 in:
Data Analysis in Community and Landscape Ecology
(Jongman, R.H.G., Ter Braak, C.J.F. and Van Tongeren, O.F.R., Eds)
Cambridge University Press, Cambridge, UK, 91-173 pp.

*** Type of analysis ***

Model	Gradient analysis		
	indirect	direct	hybrid
linear	1=PCA	2= RDA	3
unimodal	4= CA	5= CCA	6
,,	7=DCA	8=DCCA	9
	10=non-standard analysis		

Type analysis number
Answer = 2

*** Data files ***
Species data : D:\Canoco\spe.dta
Covariable data :
Environmental data : D:\Canoco\env.dta
Initialization file:

Forward selection of envi. variables = 1
Scaling of ordination scores = 2
Diagnostics = 1

File : D:\Canoco\spe.dta
Title : WCanoImp produced data file
Format : (I5,1X,5F14.9,27(/6X,(5F14.9)))
No. of couplets of species number and abundance per line : 0

No samples omitted
Number of samples 23
Number of species 137
Number of occurrences 797

File : D:\Canoco\env.dta
Title : WCanoImp produced data file
Format : (I5,1X,4F15.9,3(/6X,(4F15.9)))
No. of environmental variables : 13

No interaction terms defined

Squareroot-transformation of species data
No species-weights specified
No sample-weights specified
Centering/standardization by species = 1
Centering/standardization by samples = 0

No. of active samples: 23
No. of passive samples: 0
No. of active species: 137

Total sum of squares in species data = 366.587

Total standard deviation in species data TAU = 0.341086

****** Check on influence in covariable/environment data ******
The following sample(s) have extreme values

Sample	Environmental variable	Influence	Covariable influence	+ Environment space influence
17	7	5.2x		
18	1	8.7x		
18	3	42.6x		
18	6	5.2x		
18	8	25.7x		
18	9	8.5x		
22	5	6.5x		

****** End of check ******

**** Start of forward selection of variables ****

*** Unrestricted permutation ***

Seeds: 23239 945

N	Name	Extra fit
12	Clay	0.04
6	Phosphate	0.08
5	Nitrate	0.09
13	Mz	0.09
1	Depth	0.09
9	POC	0.09
4	Silicate	0.10
8	Chlor_a	0.11
7	Oxygen	0.11
11	Silt	0.12
3	SSS	0.13
2	SST	0.14

Environmental variable 2 tested
Number of permutations= 999

P-value 0.0040 (variable 2; F-ratio= 3.34; number of permutations= 999)

Environmental variable 2 added to model
Variance explained by the variables selected: 0.14
" " " all variables : 0.72

N	Name	Extra fit
7	Oxygen	0.04
12	Clay	0.05
13	Mz	0.09
4	Silicate	0.09
6	Phosphate	0.10
11	Silt	0.12
5	Nitrate	0.13
9	POC	0.16
1	Depth	0.18
8	Chlor_a	0.21
3	SSS	0.25

Environmental variable 3 tested
Number of permutations= 999

P-value 0.0010 (variable 3; F-ratio= 8.01; number of permutations= 999)

Environmental variable 3 added to model
Variance explained by the variables selected: 0.38
" " " all variables : 0.72

N	Name	Extra fit
6	Phosphate	0.03
8	Chlor_a	0.04
1	Depth	0.04
9	POC	0.04
12	Clay	0.04
7	Oxygen	0.04
5	Nitrate	0.04
4	Silicate	0.05
13	Mz	0.05
11	Silt	0.06

Environmental variable 11 tested
Number of permutations= 999

P-value 0.0170 (variable 11; F-ratio= 1.90; number of permutations= 999)

Environmental variable 11 added to model
Variance explained by the variables selected: 0.44
" " " all variables : 0.72

N	Name	Extra fit
1	Depth	0.03
6	Phosphate	0.03
8	Chlor_a	0.04
13	Mz	0.04
9	POC	0.04
7	Oxygen	0.04
12	Clay	0.04
5	Nitrate	0.04
4	Silicate	0.04

Environmental variable 4 tested
Number of permutations= 999

P-value 0.0720 (variable 4; F-ratio= 1.55; number of permutations= 999)

Environmental variable 4 added to model
Variance explained by the variables selected: 0.48
" " " all variables : 0.72

N	Name	Extra fit
1	Depth	0.03
8	Chlor_a	0.03
6	Phosphate	0.03
9	POC	0.03
13	Mz	0.04
7	Oxygen	0.04
12	Clay	0.04
5	Nitrate	0.04

Environmental variable 5 tested
Number of permutations= 999

P-value 0.0810 (variable 5; F-ratio= 1.47; number of permutations= 999)

Environmental variable 5 added to model

Variance explained by the variables selected: 0.53
" " " all variables : 0.72

N Name Extra fit

6 Phosphate 0.03
1 Depth 0.03
9 POC 0.03
8 Chlor_a 0.03
12 Clay 0.04
13 Mz 0.04
7 Oxygen 0.04

Environmental variable 7 tested
Number of permutations= 999

P-value 0.0610 (variable 7; F-ratio= 1.63; number of permutations= 999)

Environmental variable 7 added to model
Variance explained by the variables selected: 0.57
" " " all variables : 0.72

N Name Extra fit

13 Mz 0.02
12 Clay 0.02
8 Chlor_a 0.03
1 Depth 0.03
6 Phosphate 0.03
9 POC 0.03

Environmental variable 9 tested
Number of permutations= 999

P-value 0.3200 (variable 9; F-ratio= 1.13; number of permutations= 999)

Environmental variable 9 added to model
Variance explained by the variables selected: 0.60
" " " all variables : 0.72

N Name Extra fit

8 Chlor_a 0.02
13 Mz 0.02

12 Clay 0.02
1 Depth 0.03
6 Phosphate 0.03
Environmental variable 6 tested
Number of permutations= 999

P-value 0.2890 (variable 6; F-ratio= 1.17; number of permutations= 999)

Environmental variable 6 added to model
Variance explained by the variables selected: 0.63
" " " all variables : 0.72

N Name Extra fit

8 Chlor_a 0.02
13 Mz 0.02
12 Clay 0.02
1 Depth 0.03
Environmental variable 1 tested
Number of permutations= 999

P-value 0.4300 (variable 1; F-ratio= 1.02; number of permutations= 999)

Environmental variable 1 added to model
Variance explained by the variables selected: 0.66
" " " all variables : 0.72

N Name Extra fit

13 Mz 0.02
12 Clay 0.02
8 Chlor_a 0.02
Environmental variable 8 tested
Number of permutations= 999

P-value 0.7030 (variable 8; F-ratio= 0.78; number of permutations= 999)

Environmental variable 8 added to model
Variance explained by the variables selected: 0.68
" " " all variables : 0.72

N Name Extra fit

13 Mz 0.02
12 Clay 0.02
Environmental variable 12 tested
Number of permutations= 999

P-value 0.7170 (variable 12; F-ratio= 0.78; number of permutations= 999)

Environmental variable 12 added to model
Variance explained by the variables selected: 0.70
" " " all variables : 0.72

N Name Extra fit

13 Mz 0.02
Environmental variable 13 tested
Number of permutations= 999

P-value 0.7300 (variable 13; F-ratio= 0.72; number of permutations= 999)

Environmental variable 13 added to model
Variance explained by the variables selected: 0.72
" " " all variables : 0.72

No more variables to improve fit
*** End of selection ***

N	name	(weighted) mean	stand. dev.	inflation factor
1	SPEC AX1	0.0000	1.0182	
2	SPEC AX2	0.0000	1.0212	
3	SPEC AX3	0.0000	1.0545	
4	SPEC AX4	0.0000	1.1662	
5	ENVI AX1	0.0000	1.0000	
6	ENVI AX2	0.0000	1.0000	
7	ENVI AX3	0.0000	1.0000	
8	ENVI AX4	0.0000	1.0000	
1	Depth	74.7565	16.8072	14.5845
2	SST	21.1497	1.3663	58.0160
3	SSS	33.1070	1.5214	123.1374

4	Silicate	7.0615	1.8636	61.2506
5	Nitrate	2.0589	0.8470	30.4940
6	Phosphate	0.2368	0.0477	23.7822
7	Oxygen	5.2959	0.2116	27.0383
8	Chlor_a	1.2082	1.1019	286.7826
9	POC	168.3448	59.7897	130.6157
11	Silt	0.3901	0.1932	897.1188
12	Clay	0.1329	0.0624	256.8089
13	Mz	4.4115	1.0372	**1839.1206**

Note: "Mz" was removed from RDA model.

**** Summary ****

Axes 1 2 3 4
Total variance

Eigenvalues : 0.321 0.106 0.071 0.049
1.000
Species-environment correlations : 0.982 0.979 0.948 0.858
Cumulative percentage variance
of species data : 32.1 42.7 49.8 54.7
of species-environment relation: 44.7 59.4 69.2 76.0

Sum of all eigenvalues
1.000
Sum of all canonical eigenvalues
0.719

[Wed Jul 08 13:49:36 2020] CANOCO call succeeded
[Wed Jul 08 13:51:19 2020] Settings changed
[Wed Jul 08 13:51:20 2020] Running CANOCO:
[Wed Jul 08 13:51:20 2020] CON file [D:\Canoco\spe.con] saved
Program CANOCO Version 4.5 February 2002 - written by Cajo J.F. Ter Braak
(C) 1988-2002 Biometris - quantitative methods in the life and earth sciences
Plant Research International, Wageningen University and Research Centre
Box 100, 6700 AC Wageningen, the Netherlands
CANOCO performs (partial) (detrended) (canonical) correspondence analysis,
principal components analysis and redundancy analysis.
CANOCO is an extension of Cornell Ecology program DECORANA (Hill,1979)

For explanation of the input/output see the manual or
Ter Braak, C.J.F. (1995) Ordination. Chapter 5 in:
Data Analysis in Community and Landscape Ecology

(Jongman, R.H.G., Ter Braak, C.J.F. and Van Tongeren, O.F.R., Eds)
Cambridge University Press, Cambridge, UK, 91-173 pp.

*** Type of analysis ***

Model	Gradient analysis		
	indirect	direct	hybrid
linear	1=PCA	2= RDA	3
unimodal	4= CA	5= CCA	6
,,	7=DCA	8=DCCA	9
	10=non-standard analysis		

Type analysis number
Answer = 2

*** Data files ***
Species data : D:\Canoco\spe.dta
Covariable data :
Environmental data : D:\Canoco\env.dta
Initialization file:

Forward selection of envi. variables = 1
Scaling of ordination scores = 2
Diagnostics = 1

File : D:\Canoco\spe.dta
Title : WCanoImp produced data file
Format : (I5,1X,5F14.9,27(/6X,(5F14.9)))
No. of couplets of species number and abundance per line : 0

No samples omitted
Number of samples 23
Number of species 137
Number of occurrences 797

File : D:\Canoco\env.dta
Title : WCanoImp produced data file
Format : (I5,1X,4F15.9,3(/6X,(4F15.9)))
No. of environmental variables : 13

No interaction terms defined

Squareroot-transformation of species data
No species-weights specified
No sample-weights specified
Centering/standardization by species = 1
Centering/standardization by samples = 0

No. of active samples: 23
No. of passive samples: 0
No. of active species: 137

Total sum of squares in species data = 366.587
Total standard deviation in species data TAU = 0.341086

****** Check on influence in covariable/environment data ******
The following sample(s) have extreme values

Sample	Environmental variable	Influence	Covariable influence	+ Environment space influence
17	7	5.2x		
18	1	8.7x		
18	3	42.6x		
18	6	5.2x		
18	8	25.7x		
18	9	8.5x		
22	5	6.5x		

****** End of check ******

**** Start of forward selection of variables ****

*** Unrestricted permutation ***

Seeds: 23239 945

N	Name	Extra fit
12	Clay	0.04
6	Phosphate	0.08
5	Nitrate	0.09
1	Depth	0.09
9	POC	0.09

4 Silicate 0.10
8 Chlor_a 0.11
7 Oxygen 0.11
11 Silt 0.12
3 SSS 0.13
2 SST 0.14

Environmental variable 2 tested
Number of permutations= 999

P-value 0.0040 (variable 2; F-ratio= 3.34; number of permutations= 999)

Environmental variable 2 added to model
Variance explained by the variables selected: 0.14
" " " all variables : 0.70

N Name Extra fit

7 Oxygen 0.04
12 Clay 0.05
4 Silicate 0.09
6 Phosphate 0.10
11 Silt 0.12
5 Nitrate 0.13
9 POC 0.16
1 Depth 0.18
8 Chlor_a 0.21
3 SSS 0.25

Environmental variable 3 tested
Number of permutations= 999

P-value 0.0010 (variable 3; F-ratio= 8.01; number of permutations= 999)

Environmental variable 3 added to model
Variance explained by the variables selected: 0.38
" " " all variables : 0.70

N Name Extra fit

6 Phosphate 0.03
8 Chlor_a 0.04
1 Depth 0.04
9 POC 0.04

12 Clay 0.04
7 Oxygen 0.04
5 Nitrate 0.04
4 Silicate 0.05
11 Silt 0.06
Environmental variable 11 tested
Number of permutations= 999

P-value 0.0170 (variable 11; F-ratio= 1.90; number of permutations= 999)

Environmental variable 11 added to model
Variance explained by the variables selected: 0.44
" " " all variables : 0.70

N Name Extra fit

1 Depth 0.03
6 Phosphate 0.03
8 Chlor_a 0.04
9 POC 0.04
7 Oxygen 0.04
12 Clay 0.04
5 Nitrate 0.04
4 Silicate 0.04
Environmental variable 4 tested
Number of permutations= 999

P-value 0.0720 (variable 4; F-ratio= 1.55; number of permutations= 999)

Environmental variable 4 added to model
Variance explained by the variables selected: 0.48
" " " all variables : 0.70

N Name Extra fit

1 Depth 0.03
8 Chlor_a 0.03
6 Phosphate 0.03
9 POC 0.03
7 Oxygen 0.04
12 Clay 0.04
5 Nitrate 0.04

Environmental variable 5 tested
Number of permutations= 999

P-value 0.0810 (variable 5; F-ratio= 1.47; number of permutations= 999)

Environmental variable 5 added to model
Variance explained by the variables selected: 0.53
" " " all variables : 0.70

N Name Extra fit

6 Phosphate 0.03
1 Depth 0.03
9 POC 0.03
8 Chlor_a 0.03
12 Clay 0.04
7 Oxygen 0.04

Environmental variable 7 tested
Number of permutations= 999

P-value 0.0610 (variable 7; F-ratio= 1.63; number of permutations= 999)

Environmental variable 7 added to model
Variance explained by the variables selected: 0.57
" " " all variables : 0.70

N Name Extra fit

12 Clay 0.02
8 Chlor_a 0.03
1 Depth 0.03
6 Phosphate 0.03
9 POC 0.03

Environmental variable 9 tested
Number of permutations= 999

P-value 0.3200 (variable 9; F-ratio= 1.13; number of permutations= 999)

Environmental variable 9 added to model
Variance explained by the variables selected: 0.60
" " " all variables : 0.70

N Name Extra fit

8 Chlor_a 0.02
12 Clay 0.02
1 Depth 0.03
6 Phosphate 0.03
Environmental variable 6 tested
Number of permutations= 999

P-value 0.2890 (variable 6; F-ratio= 1.17; number of permutations= 999)

Environmental variable 6 added to model
Variance explained by the variables selected: 0.63
" " " all variables : 0.70

N Name Extra fit

8 Chlor_a 0.02
12 Clay 0.02
1 Depth 0.03
Environmental variable 1 tested
Number of permutations= 999

P-value 0.4300 (variable 1; F-ratio= 1.02; number of permutations= 999)

Environmental variable 1 added to model
Variance explained by the variables selected: 0.66
" " " all variables : 0.70

N Name Extra fit

12 Clay 0.02
8 Chlor_a 0.02
Environmental variable 8 tested
Number of permutations= 999

P-value 0.7030 (variable 8; F-ratio= 0.78; number of permutations= 999)

Environmental variable 8 added to model
Variance explained by the variables selected: 0.68

" " " all variables : 0.70

N Name Extra fit

12 Clay 0.02
Environmental variable 12 tested
Number of permutations= 999

P-value 0.7170 (variable 12; F-ratio= 0.78; number of permutations= 999)

Environmental variable 12 added to model
Variance explained by the variables selected: 0.70
" " " all variables : 0.70

No more variables to improve fit
*** End of selection ***

N	name	(weighted) mean	stand. dev.	inflation factor
1	SPEC AX1	0.0000	1.0186	
2	SPEC AX2	0.0000	1.0212	
3	SPEC AX3	0.0000	1.0547	
4	SPEC AX4	0.0000	1.1657	
5	ENVI AX1	0.0000	1.0000	
6	ENVI AX2	0.0000	1.0000	
7	ENVI AX3	0.0000	1.0000	
8	ENVI AX4	0.0000	1.0000	
1	Depth	74.7565	16.8072	14.5177
2	SST	21.1497	1.3663	57.6240
3	SSS	33.1070	1.5214	110.6693
4	Silicate	7.0615	1.8636	51.8251
5	Nitrate	2.0589	0.8470	10.4252
6	Phosphate	0.2368	0.0477	23.7788
7	Oxygen	5.2959	0.2116	21.9723
8	Chlor_a	1.2082	1.1019	**257.8645**
9	POC	168.3448	59.7897	94.0426
11	Silt	0.3901	0.1932	20.4520
12	Clay	0.1329	0.0624	14.5212

Note: “Chlor_a” was removed from RDA model.

**** Summary ****

Axes 1 2 3 4
Total variance

Eigenvalues : 0.321 0.106 0.071 0.048
1.000
Species-environment correlations : 0.982 0.979 0.948 0.858
Cumulative percentage variance
of species data : 32.1 42.7 49.8 54.6
of species-environment relation: 45.9 61.0 71.2 78.1

Sum of all eigenvalues
1.000
Sum of all canonical eigenvalues
0.699

[Wed Jul 08 13:51:24 2020] CANOCO call succeeded
[Wed Jul 08 13:51:50 2020] Settings changed
[Wed Jul 08 13:51:51 2020] Running CANOCO:
[Wed Jul 08 13:51:51 2020] CON file [D:\Canoco\spe.con] saved
Program CANOCO Version 4.5 February 2002 - written by Cajo J.F. Ter Braak
(C) 1988-2002 Biometris - quantitative methods in the life and earth sciences
Plant Research International, Wageningen University and Research Centre
Box 100, 6700 AC Wageningen, the Netherlands
CANOCO performs (partial) (detrended) (canonical) correspondence analysis,
principal components analysis and redundancy analysis.
CANOCO is an extension of Cornell Ecology program DECORANA (Hill,1979)

For explanation of the input/output see the manual or
Ter Braak, C.J.F. (1995) Ordination. Chapter 5 in:
Data Analysis in Community and Landscape Ecology
(Jongman, R.H.G., Ter Braak, C.J.F. and Van Tongeren, O.F.R., Eds)
Cambridge University Press, Cambridge, UK, 91-173 pp.

*** Type of analysis ***

Model	Gradient analysis		
	indirect	direct	hybrid
linear	1=PCA	2= RDA	3
unimodal	4= CA	5= CCA	6
,,	7=DCA	8=DCCA	9
	10=non-standard analysis		

Type analysis number
Answer = 2

*** Data files ***
Species data : D:\Canoco\spe.dta
Covariable data :
Environmental data : D:\Canoco\env.dta
Initialization file:

Forward selection of envi. variables = 1
Scaling of ordination scores = 2
Diagnostics = 1

File : D:\Canoco\spe.dta
Title : WCanoImp produced data file
Format : (I5,1X,5F14.9,27(/6X,(5F14.9)))
No. of couplets of species number and abundance per line : 0

No samples omitted
Number of samples 23
Number of species 137
Number of occurrences 797

File : D:\Canoco\env.dta
Title : WCanoImp produced data file
Format : (I5,1X,4F15.9,3(/6X,(4F15.9)))
No. of environmental variables : 13

No interaction terms defined

Squareroot-transformation of species data
No species-weights specified
No sample-weights specified
Centering/standardization by species = 1
Centering/standardization by samples = 0

No. of active samples: 23
No. of passive samples: 0
No. of active species: 137

Total sum of squares in species data = 366.587
Total standard deviation in species data TAU = 0.341086

****** Check on influence in covariable/environment data ******
The following sample(s) have extreme values

Sample	Environmental variable	Influence	Covariable influence	+ Environment space influence
17	7	5.2x		
18	1	8.7x		
18	3	42.6x		
18	6	5.2x		
18	9	8.5x		
22	5	6.5x		

****** End of check ******

**** Start of forward selection of variables ****

*** Unrestricted permutation ***

Seeds: 23239 945

N	Name	Extra fit
12	Clay	0.04
6	Phosphate	0.08
5	Nitrate	0.09
1	Depth	0.09
9	POC	0.09
4	Silicate	0.10
7	Oxygen	0.11
11	Silt	0.12
3	SSS	0.13
2	SST	0.14

Environmental variable 2 tested
Number of permutations= 999

P-value 0.0040 (variable 2; F-ratio= 3.34; number of permutations= 999)

Environmental variable 2 added to model
Variance explained by the variables selected: 0.14
" " " all variables : 0.68

N	Name	Extra fit
7	Oxygen	0.04
12	Clay	0.05
4	Silicate	0.09
6	Phosphate	0.10
11	Silt	0.12
5	Nitrate	0.13
9	POC	0.16
1	Depth	0.18
3	SSS	0.25

Environmental variable 3 tested
Number of permutations= 999

P-value 0.0010 (variable 3; F-ratio= 8.01; number of permutations= 999)

Environmental variable 3 added to model
Variance explained by the variables selected: 0.38
" " " all variables : 0.68

N	Name	Extra fit
6	Phosphate	0.03
1	Depth	0.04
9	POC	0.04
12	Clay	0.04
7	Oxygen	0.04
5	Nitrate	0.04
4	Silicate	0.05
11	Silt	0.06

Environmental variable 11 tested
Number of permutations= 999

P-value 0.0170 (variable 11; F-ratio= 1.90; number of permutations= 999)

Environmental variable 11 added to model
Variance explained by the variables selected: 0.44
" " " all variables : 0.68

N Name Extra fit

1 Depth 0.03
6 Phosphate 0.03
9 POC 0.04
7 Oxygen 0.04
12 Clay 0.04
5 Nitrate 0.04
4 Silicate 0.04
Environmental variable 4 tested
Number of permutations= 999

P-value 0.0720 (variable 4; F-ratio= 1.55; number of permutations= 999)

Environmental variable 4 added to model
Variance explained by the variables selected: 0.48
" " " all variables : 0.68

N Name Extra fit

1 Depth 0.03
6 Phosphate 0.03
9 POC 0.03
7 Oxygen 0.04
12 Clay 0.04
5 Nitrate 0.04
Environmental variable 5 tested
Number of permutations= 999

P-value 0.0810 (variable 5; F-ratio= 1.47; number of permutations= 999)

Environmental variable 5 added to model
Variance explained by the variables selected: 0.53
" " " all variables : 0.68

N Name Extra fit

6 Phosphate 0.03
1 Depth 0.03
9 POC 0.03
12 Clay 0.04
7 Oxygen 0.04
Environmental variable 7 tested
Number of permutations= 999

P-value 0.0610 (variable 7; F-ratio= 1.63; number of permutations= 999)

Environmental variable 7 added to model
Variance explained by the variables selected: 0.57
" " " all variables : 0.68

N Name Extra fit

12 Clay 0.02
1 Depth 0.03
6 Phosphate 0.03
9 POC 0.03
Environmental variable 9 tested
Number of permutations= 999

P-value 0.3200 (variable 9; F-ratio= 1.13; number of permutations= 999)

Environmental variable 9 added to model
Variance explained by the variables selected: 0.60
" " " all variables : 0.68

N Name Extra fit

12 Clay 0.02
1 Depth 0.03
6 Phosphate 0.03
Environmental variable 6 tested
Number of permutations= 999

P-value 0.2890 (variable 6; F-ratio= 1.17; number of permutations= 999)

Environmental variable 6 added to model
Variance explained by the variables selected: 0.63
" " " all variables : 0.68

N Name Extra fit

12 Clay 0.02
1 Depth 0.03
Environmental variable 1 tested

Number of permutations= 999

P-value 0.4300 (variable 1; F-ratio= 1.02; number of permutations= 999)

Environmental variable 1 added to model
Variance explained by the variables selected: 0.66
" " " all variables : 0.68

N Name Extra fit

12 Clay 0.02
Environmental variable 12 tested
Number of permutations= 999

P-value 0.7470 (variable 12; F-ratio= 0.73; number of permutations= 999)

Environmental variable 12 added to model
Variance explained by the variables selected: 0.68
" " " all variables : 0.68

No more variables to improve fit
*** End of selection ***

N	name	(weighted) mean	stand. dev.	inflation factor
1	SPEC AX1	0.0000	1.0207	
2	SPEC AX2	0.0000	1.0221	
3	SPEC AX3	0.0000	1.0542	
4	SPEC AX4	0.0000	1.1701	
5	ENVI AX1	0.0000	1.0000	
6	ENVI AX2	0.0000	1.0000	
7	ENVI AX3	0.0000	1.0000	
8	ENVI AX4	0.0000	1.0000	
1	Depth	74.7565	16.8072	11.7253
2	SST	21.1497	1.3663	16.5240
3	SSS	33.1070	1.5214	12.1269
4	Silicate	7.0615	1.8636	**49.9732**
5	Nitrate	2.0589	0.8470	8.8455
6	Phosphate	0.2368	0.0477	22.9917
7	Oxygen	5.2959	0.2116	17.0672
9	POC	168.3448	59.7897	35.0952

11	Silt	0.3901	0.1932	14.2892
12	Clay	0.1329	0.0624	11.0195

**** Summary ****

Note: "Silicate" was removed from RDA model.

Axes	1	2	3	4	Total variance
Eigenvalues :	0.320	0.105	0.071	0.048	1.000
Species-environment correlations :	0.980	0.978	0.949	0.855	
Cumulative percentage variance					
of species data :	32.0	42.5	49.6	54.4	
of species-environment relation:	47.2	62.8	73.3	80.4	

Sum of all eigenvalues 1.000
Sum of all canonical eigenvalues 0.677

[Wed Jul 08 13:51:54 2020] CANOCO call succeeded
[Wed Jul 08 13:52:20 2020] Settings changed
[Wed Jul 08 13:52:22 2020] Running CANOCO:
[Wed Jul 08 13:52:22 2020] CON file [D:\Canoco\spe.con] saved
Program CANOCO Version 4.5 February 2002 - written by Cajo J.F. Ter Braak
(C) 1988-2002 Biometris - quantitative methods in the life and earth sciences
Plant Research International, Wageningen University and Research Centre
Box 100, 6700 AC Wageningen, the Netherlands
CANOCO performs (partial) (detrended) (canonical) correspondence analysis,
principal components analysis and redundancy analysis.
CANOCO is an extension of Cornell Ecology program DECORANA (Hill,1979)

For explanation of the input/output see the manual or
Ter Braak, C.J.F. (1995) Ordination. Chapter 5 in:
Data Analysis in Community and Landscape Ecology
(Jongman, R.H.G., Ter Braak, C.J.F. and Van Tongeren, O.F.R., Eds)
Cambridge University Press, Cambridge, UK, 91-173 pp.

*** Type of analysis ***

Model	Gradient analysis		
	indirect	direct	hybrid

linear 1=PCA 2= RDA 3
unimodal 4= CA 5= CCA 6
,, 7=DCA 8=DCCA 9
10=non-standard analysis
Type analysis number
Answer = 2

*** Data files ***
Species data : D:\Canoco\spe.dta
Covariable data :
Environmental data : D:\Canoco\env.dta
Initialization file:

Forward selection of envi. variables = 1
Scaling of ordination scores = 2
Diagnostics = 1

File : D:\Canoco\spe.dta
Title : WCanoImp produced data file
Format : (I5,1X,5F14.9,27(/6X,(5F14.9)))
No. of couplets of species number and abundance per line : 0

No samples omitted
Number of samples 23
Number of species 137
Number of occurrences 797

File : D:\Canoco\env.dta
Title : WCanoImp produced data file
Format : (I5,1X,4F15.9,3(/6X,(4F15.9)))
No. of environmental variables : 13

No interaction terms defined

Squareroot-transformation of species data
No species-weights specified
No sample-weights specified
Centering/standardization by species = 1
Centering/standardization by samples = 0

No. of active samples: 23
No. of passive samples: 0
No. of active species: 137

Total sum of squares in species data = 366.587
Total standard deviation in species data TAU = 0.341086

****** Check on influence in covariable/environment data ******
The following sample(s) have extreme values

Sample	Environmental variable	Influence	Covariable influence	+ Environment space influence
17	7	5.2x		
18	1	8.7x		
18	3	42.6x		
18	6	5.2x		
18	9	8.5x		
22	5	6.5x		

****** End of check ******

**** Start of forward selection of variables ****

*** Unrestricted permutation ***

Seeds: 23239 945

N	Name	Extra fit
12	Clay	0.04
6	Phosphate	0.08
5	Nitrate	0.09
1	Depth	0.09
9	POC	0.09
7	Oxygen	0.11
11	Silt	0.12
3	SSS	0.13
2	SST	0.14

Environmental variable 2 tested
Number of permutations= 999

P-value 0.0040 (variable 2; F-ratio= 3.34; number of permutations= 999)

Environmental variable 2 added to model
Variance explained by the variables selected: 0.14
" " " all variables : 0.65

N	Name	Extra fit
7	Oxygen	0.04
12	Clay	0.05
6	Phosphate	0.10
11	Silt	0.12
5	Nitrate	0.13
9	POC	0.16
1	Depth	0.18
3	SSS	0.25

Environmental variable 3 tested
Number of permutations= 999

P-value 0.0010 (variable 3; F-ratio= 8.01; number of permutations= 999)

Environmental variable 3 added to model
Variance explained by the variables selected: 0.38
" " " all variables : 0.65

N	Name	Extra fit
6	Phosphate	0.03
1	Depth	0.04
9	POC	0.04
12	Clay	0.04
7	Oxygen	0.04
5	Nitrate	0.04
11	Silt	0.06

Environmental variable 11 tested
Number of permutations= 999

P-value 0.0170 (variable 11; F-ratio= 1.90; number of permutations= 999)

Environmental variable 11 added to model
Variance explained by the variables selected: 0.44

"   "   "   all variables   :   0.65

N   Name Extra fit

1 Depth   0.03
6 Phosphate   0.03
9 POC   0.04
7 Oxygen   0.04
12 Clay   0.04
5 Nitrate   0.04

Environmental variable   5 tested
Number of permutations=   999

P-value 0.0690 (variable   5; F-ratio=   1.54; number of permutations=   999)

Environmental variable   5 added to model
Variance explained by the variables selected: 0.48
"   "   "   all variables   :   0.65

N   Name Extra fit

1 Depth   0.03
9 POC   0.03
6 Phosphate   0.04
12 Clay   0.04
7 Oxygen   0.04

Environmental variable   7 tested
Number of permutations=   999

P-value 0.0900 (variable   7; F-ratio=   1.52; number of permutations=   999)

Environmental variable   7 added to model
Variance explained by the variables selected: 0.53
"   "   "   all variables   :   0.65

N   Name Extra fit

1 Depth   0.03
12 Clay   0.03
9 POC   0.03
6 Phosphate   0.04

Environmental variable   6 tested

Number of permutations= 999

P-value 0.0850 (variable 6; F-ratio= 1.47; number of permutations= 999)

Environmental variable 6 added to model
Variance explained by the variables selected: 0.57
" " " all variables : 0.65

N Name Extra fit

12 Clay 0.03
1 Depth 0.03
9 POC 0.03
Environmental variable 9 tested
Number of permutations= 999

P-value 0.1690 (variable 9; F-ratio= 1.31; number of permutations= 999)

Environmental variable 9 added to model
Variance explained by the variables selected: 0.60
" " " all variables : 0.65

N Name Extra fit

12 Clay 0.02
1 Depth 0.03
Environmental variable 1 tested
Number of permutations= 999

P-value 0.4240 (variable 1; F-ratio= 1.03; number of permutations= 999)

Environmental variable 1 added to model
Variance explained by the variables selected: 0.63
" " " all variables : 0.65

N Name Extra fit

12 Clay 0.02
Environmental variable 12 tested
Number of permutations= 999

P-value 0.8010 (variable 12; F-ratio= 0.70; number of permutations= 999)

Environmental variable 12 added to model
Variance explained by the variables selected: 0.65
" " " all variables : 0.65

No more variables to improve fit
*** End of selection ***

N	name	(weighted) mean	stand. dev.	inflation factor
1	SPEC AX1	0.0000	1.0210	
2	SPEC AX2	0.0000	1.0291	
3	SPEC AX3	0.0000	1.0686	
4	SPEC AX4	0.0000	1.1787	
5	ENVI AX1	0.0000	1.0000	
6	ENVI AX2	0.0000	1.0000	
7	ENVI AX3	0.0000	1.0000	
8	ENVI AX4	0.0000	1.0000	
1	Depth	74.7565	16.8072	10.1678
2	SST	21.1497	1.3663	13.3485
3	SSS	33.1070	1.5214	8.6591
5	Nitrate	2.0589	0.8470	8.7642
6	Phosphate	0.2368	0.0477	4.8862
7	Oxygen	5.2959	0.2116	17.0049
9	POC	168.3448	59.7897	**20.9039**
11	Silt	0.3901	0.1932	12.7067
12	Clay	0.1329	0.0624	10.1297

Note: "POC" was removed from RDA model.

**** Summary ****

Axes	1	2	3	4	Total variance
Eigenvalues :	0.319	0.103	0.069	0.048	1.000
Species-environment correlations :	0.979	0.972	0.936	0.848	
Cumulative percentage variance					
of species data :	31.9	42.2	49.2	54.0	
of species-environment relation:	49.3	65.2	75.9	83.4	

Sum of all eigenvalues
1.000
Sum of all canonical eigenvalues
0.647

[Wed Jul 08 13:52:25 2020] CANOCO call succeeded
[Wed Jul 08 13:54:15 2020] Settings changed
[Wed Jul 08 13:54:16 2020] Running CANOCO:
[Wed Jul 08 13:54:16 2020] CON file [D:\Canoco\spe.con] saved
Program CANOCO Version 4.5 February 2002 - written by Cajo J.F. Ter Braak
(C) 1988-2002 Biometris - quantitative methods in the life and earth sciences
Plant Research International, Wageningen University and Research Centre
Box 100, 6700 AC Wageningen, the Netherlands
CANOCO performs (partial) (detrended) (canonical) correspondence analysis,
principal components analysis and redundancy analysis.
CANOCO is an extension of Cornell Ecology program DECORANA (Hill,1979)

For explanation of the input/output see the manual or
Ter Braak, C.J.F. (1995) Ordination. Chapter 5 in:
Data Analysis in Community and Landscape Ecology
(Jongman, R.H.G., Ter Braak, C.J.F. and Van Tongeren, O.F.R., Eds)
Cambridge University Press, Cambridge, UK, 91-173 pp.

*** Type of analysis ***

Model	Gradient analysis		
	indirect	direct	hybrid
linear	1=PCA	2= RDA	3
unimodal	4= CA	5= CCA	6
,,	7=DCA	8=DCCA	9
	10=non-standard analysis		

Type analysis number
Answer = 2

*** Data files ***
Species data : D:\Canoco\spe.dta
Covariable data :
Environmental data : D:\Canoco\env.dta
Initialization file:

Forward selection of envi. variables = 1
Scaling of ordination scores = 2
Diagnostics = 1

File : D:\Canoco\spe.dta
Title : WCanoImp produced data file
Format : (I5,1X,5F14.9,27(/6X,(5F14.9)))
No. of couplets of species number and abundance per line : 0

No samples omitted
Number of samples 23
Number of species 137
Number of occurrences 797

File : D:\Canoco\env.dta
Title : WCanoImp produced data file
Format : (I5,1X,4F15.9,3(/6X,(4F15.9)))
No. of environmental variables : 13

No interaction terms defined

Squareroot-transformation of species data
No species-weights specified
No sample-weights specified
Centering/standardization by species = 1
Centering/standardization by samples = 0

No. of active samples: 23
No. of passive samples: 0
No. of active species: 137

Total sum of squares in species data = 366.587
Total standard deviation in species data TAU = 0.341086

****** Check on influence in covariable/environment data ******
The following sample(s) have extreme values

Sample	Environmental variable	Influence	Covariable influence	+ Environment space influence
17	7	5.2x		
18	1	8.7x		
18	3	42.6x		
18	6	5.2x		

22 5 6.5x
****** End of check ******

**** Start of forward selection of variables ****

*** Unrestricted permutation ***

Seeds: 23239 945

N	Name	Extra fit
12	Clay	0.04
6	Phosphate	0.08
5	Nitrate	0.09
1	Depth	0.09
7	Oxygen	0.11
11	Silt	0.12
3	SSS	0.13
2	SST	0.14

Environmental variable 2 tested
Number of permutations= 999

P-value 0.0040 (variable 2; F-ratio= 3.34; number of permutations= 999)

Environmental variable 2 added to model
Variance explained by the variables selected: 0.14
" " " all variables : 0.62

N	Name	Extra fit
7	Oxygen	0.04
12	Clay	0.05
6	Phosphate	0.10
11	Silt	0.12
5	Nitrate	0.13
1	Depth	0.18
3	SSS	0.25

Environmental variable 3 tested
Number of permutations= 999

P-value 0.0010 (variable 3; F-ratio= 8.01; number of permutations= 999)

Environmental variable 3 added to model
Variance explained by the variables selected: 0.38
" " " all variables : 0.62

N Name Extra fit

6 Phosphate 0.03
1 Depth 0.04
12 Clay 0.04
7 Oxygen 0.04
5 Nitrate 0.04
11 Silt 0.06
Environmental variable 11 tested
Number of permutations= 999

P-value 0.0170 (variable 11; F-ratio= 1.90; number of permutations= 999)

Environmental variable 11 added to model
Variance explained by the variables selected: 0.44
" " " all variables : 0.62

N Name Extra fit

1 Depth 0.03
6 Phosphate 0.03
7 Oxygen 0.04
12 Clay 0.04
5 Nitrate 0.04
Environmental variable 5 tested
Number of permutations= 999

P-value 0.0690 (variable 5; F-ratio= 1.54; number of permutations= 999)

Environmental variable 5 added to model
Variance explained by the variables selected: 0.48
" " " all variables : 0.62

N Name Extra fit

1 Depth 0.03
6 Phosphate 0.04
12 Clay 0.04
7 Oxygen 0.04
Environmental variable 7 tested
Number of permutations= 999

P-value 0.0900 (variable 7; F-ratio= 1.52; number of permutations= 999)

Environmental variable 7 added to model
Variance explained by the variables selected: 0.53
" " " all variables : 0.62

N Name Extra fit

1 Depth 0.03
12 Clay 0.03
6 Phosphate 0.04
Environmental variable 6 tested
Number of permutations= 999

P-value 0.0850 (variable 6; F-ratio= 1.47; number of permutations= 999)

Environmental variable 6 added to model
Variance explained by the variables selected: 0.57
" " " all variables : 0.62

N Name Extra fit

12 Clay 0.03
1 Depth 0.03
Environmental variable 1 tested
Number of permutations= 999

P-value 0.4570 (variable 1; F-ratio= 0.99; number of permutations= 999)

Environmental variable 1 added to model
Variance explained by the variables selected: 0.59
" " " all variables : 0.62

N Name Extra fit

12 Clay 0.02
Environmental variable 12 tested
Number of permutations= 999

P-value 0.5470 (variable 12; F-ratio= 0.91; number of permutations= 999)

Environmental variable 12 added to model
Variance explained by the variables selected: 0.62
" " " all variables : 0.62

No more variables to improve fit
*** End of selection ***

N	name	(weighted) mean	stand. dev.	inflation factor
1	SPEC AX1	0.0000	1.0245	
2	SPEC AX2	0.0000	1.0286	
3	SPEC AX3	0.0000	1.0669	
4	SPEC AX4	0.0000	1.1904	
5	ENVI AX1	0.0000	1.0000	
6	ENVI AX2	0.0000	1.0000	
7	ENVI AX3	0.0000	1.0000	
8	ENVI AX4	0.0000	1.0000	
1	Depth	74.7565	16.8072	7.0435
2	SST	21.1497	1.3663	12.6453
3	SSS	33.1070	1.5214	6.1633
5	Nitrate	2.0589	0.8470	6.7992
6	Phosphate	0.2368	0.0477	4.8774
7	Oxygen	5.2959	0.2116	**16.7016**
11	Silt	0.3901	0.1932	10.3918
12	Clay	0.1329	0.0624	8.1351

Note: “Oxygen” was removed from RDA model.

**** Summary ****

Axes		1	2	3	4	Total variance
Eigenvalues	:	0.316	0.103	0.069	0.045	1.000

Species-environment correlations : 0.976 0.972 0.937 0.840
Cumulative percentage variance
of species data : 31.6 41.9 48.8 53.3
of species-environment relation: 51.2 67.8 79.0 86.3

Sum of all eigenvalues
1.000
Sum of all canonical eigenvalues
0.618

[Wed Jul 08 13:54:19 2020] CANOCO call succeeded
[Wed Jul 08 13:55:00 2020] Settings changed
[Wed Jul 08 13:55:02 2020] Running CANOCO:
[Wed Jul 08 13:55:02 2020] CON file [D:\Canoco\spe.con] saved
Program CANOCO Version 4.5 February 2002 - written by Cajo J.F. Ter Braak
(C) 1988-2002 Biometris - quantitative methods in the life and earth sciences
Plant Research International, Wageningen University and Research Centre
Box 100, 6700 AC Wageningen, the Netherlands
CANOCO performs (partial) (detrended) (canonical) correspondence analysis,
principal components analysis and redundancy analysis.
CANOCO is an extension of Cornell Ecology program DECORANA (Hill,1979)

For explanation of the input/output see the manual or
Ter Braak, C.J.F. (1995) Ordination. Chapter 5 in:
Data Analysis in Community and Landscape Ecology
(Jongman, R.H.G., Ter Braak, C.J.F. and Van Tongeren, O.F.R., Eds)
Cambridge University Press, Cambridge, UK, 91-173 pp.

*** Type of analysis ***

Model	Gradient analysis		
	indirect	direct	hybrid
linear	1=PCA	2= RDA	3
unimodal	4= CA	5= CCA	6
,,	7=DCA	8=DCCA	9
	10=non-standard analysis		

Type analysis number
Answer = 2

*** Data files ***
Species data : D:\Canoco\spe.dta
Covariable data :
Environmental data : D:\Canoco\env.dta
Initialization file:

Forward selection of envi. variables = 1
Scaling of ordination scores = 2
Diagnostics = 1

File : D:\Canoco\spe.dta
Title : WCanoImp produced data file
Format : (I5,1X,5F14.9,27(/6X,(5F14.9)))
No. of couplets of species number and abundance per line : 0

No samples omitted
Number of samples 23
Number of species 137
Number of occurrences 797

File : D:\Canoco\env.dta
Title : WCanoImp produced data file
Format : (I5,1X,4F15.9,3(/6X,(4F15.9)))
No. of environmental variables : 13

No interaction terms defined

Squareroot-transformation of species data
No species-weights specified
No sample-weights specified
Centering/standardization by species = 1
Centering/standardization by samples = 0

No. of active samples: 23
No. of passive samples: 0
No. of active species: 137

Total sum of squares in species data = 366.587
Total standard deviation in species data TAU = 0.341086

****** Check on influence in covariable/environment data ******
The following sample(s) have extreme values

Sample	Environmental variable Influence	Covariable influence	+ Environment space influence

18	1	8.7x
18	3	42.6x
18	6	5.2x
22	5	6.5x

****** End of check ******

**** Start of forward selection of variables ****

*** Unrestricted permutation ***

Seeds: 23239 945

N	Name	Extra fit
12	Clay	0.04
6	Phosphate	0.08
5	Nitrate	0.09
1	Depth	0.09
11	Silt	0.12
3	SSS	0.13
2	SST	0.14

Environmental variable 2 tested
Number of permutations= 999

P-value 0.0040 (variable 2; F-ratio= 3.34; number of permutations= 999)

Environmental variable 2 added to model
Variance explained by the variables selected: 0.14
" " " all variables : 0.59

N	Name	Extra fit
12	Clay	0.05
6	Phosphate	0.10
11	Silt	0.12
5	Nitrate	0.13
1	Depth	0.18
3	SSS	0.25

Environmental variable 3 tested
Number of permutations= 999

P-value 0.0010 (variable 3; F-ratio= 8.01; number of permutations= 999)

Environmental variable 3 added to model
Variance explained by the variables selected: 0.38
" " " all variables : 0.59

N	Name	Extra fit
6	Phosphate	0.03
1	Depth	0.04
12	Clay	0.04
5	Nitrate	0.04
11	Silt	0.06

Environmental variable 11 tested
Number of permutations= 999

P-value 0.0170 (variable 11; F-ratio= 1.90; number of permutations= 999)

Environmental variable 11 added to model
Variance explained by the variables selected: 0.44
" " " all variables : 0.59

N	Name	Extra fit
1	Depth	0.03
6	Phosphate	0.03
12	Clay	0.04
5	Nitrate	0.04

Environmental variable 5 tested
Number of permutations= 999

P-value 0.0690 (variable 5; F-ratio= 1.54; number of permutations= 999)

Environmental variable 5 added to model
Variance explained by the variables selected: 0.48
" " " all variables : 0.59

N	Name	Extra fit
1	Depth	0.03

6 Phosphate 0.04
12 Clay 0.04
Environmental variable 12 tested
Number of permutations= 999

P-value 0.0940 (variable 12; F-ratio= 1.50; number of permutations= 999)

Environmental variable 12 added to model
Variance explained by the variables selected: 0.53
" " " all variables : 0.59

N Name Extra fit

1 Depth 0.02
6 Phosphate 0.04
Environmental variable 6 tested
Number of permutations= 999

P-value 0.1180 (variable 6; F-ratio= 1.39; number of permutations= 999)

Environmental variable 6 added to model
Variance explained by the variables selected: 0.56
" " " all variables : 0.59

N Name Extra fit

1 Depth 0.03
Environmental variable 1 tested
Number of permutations= 999

P-value 0.4700 (variable 1; F-ratio= 0.97; number of permutations= 999)

Environmental variable 1 added to model
Variance explained by the variables selected: 0.59
" " " all variables : 0.59

No more variables to improve fit
*** End of selection ***

N name (weighted) mean stand. dev. inflation factor

1	SPEC AX1	0.0000	1.0255	
2	SPEC AX2	0.0000	1.0342	
3	SPEC AX3	0.0000	1.1303	
4	SPEC AX4	0.0000	1.1837	
5	ENVI AX1	0.0000	1.0000	
6	ENVI AX2	0.0000	1.0000	
7	ENVI AX3	0.0000	1.0000	
8	ENVI AX4	0.0000	1.0000	
1	Depth	74.7565	16.8072	**7.0398**
2	SST	21.1497	1.3663	4.2084
3	SSS	33.1070	1.5214	5.3986
5	Nitrate	2.0589	0.8470	5.8309
6	Phosphate	0.2368	0.0477	4.4523
11	Silt	0.3901	0.1932	6.5596
12	Clay	0.1329	0.0624	5.2944

Note: "Depth" was removed from RDA model.

**** Summary ****

Axes 1 2 3 4
Total variance

Eigenvalues : 0.316 0.100 0.064 0.042
1.000
Species-environment correlations : 0.975 0.967 0.885 0.845
Cumulative percentage variance
of species data : 31.6 41.6 47.9 52.2
of species-environment relation: 53.5 70.4 81.2 88.4

Sum of all eigenvalues
1.000
Sum of all canonical eigenvalues
0.590

[Wed Jul 08 13:55:05 2020] CANOCO call succeeded
[Wed Jul 08 13:55:25 2020] Settings changed
[Wed Jul 08 13:55:26 2020] Running CANOCO:
[Wed Jul 08 13:55:26 2020] CON file [D:\Canoco\spe.con] saved
Program CANOCO Version 4.5 February 2002 - written by Cajo J.F. Ter Braak
(C) 1988-2002 Biometris - quantitative methods in the life and earth sciences
Plant Research International, Wageningen University and Research Centre
Box 100, 6700 AC Wageningen, the Netherlands

CANOCO performs (partial) (detrended) (canonical) correspondence analysis,
principal components analysis and redundancy analysis.
CANOCO is an extension of Cornell Ecology program DECORANA (Hill,1979)

For explanation of the input/output see the manual or
Ter Braak, C.J.F. (1995) Ordination. Chapter 5 in:
Data Analysis in Community and Landscape Ecology
(Jongman, R.H.G., Ter Braak, C.J.F. and Van Tongeren, O.F.R., Eds)
Cambridge University Press, Cambridge, UK, 91-173 pp.

*** Type of analysis ***

Model	Gradient analysis		
	indirect	direct	hybrid
linear	1=PCA	2= RDA	3
unimodal	4= CA	5= CCA	6
,,	7=DCA	8=DCCA	9
	10=non-standard analysis		

Type analysis number
Answer = 2

*** Data files ***
Species data : D:\Canoco\spe.dta
Covariable data :
Environmental data : D:\Canoco\env.dta
Initialization file:

Forward selection of envi. variables = 1
Scaling of ordination scores = 2
Diagnostics = 1

File : D:\Canoco\spe.dta
Title : WCanoImp produced data file
Format : (I5,1X,5F14.9,27(/6X,(5F14.9)))
No. of couplets of species number and abundance per line : 0

No samples omitted
Number of samples 23
Number of species 137
Number of occurrences 797

File : D:\Canoco\env.dta

Title : WCanoImp produced data file
Format : (I5,1X,4F15.9,3(/6X,(4F15.9)))
No. of environmental variables : 13

No interaction terms defined

Squareroot-transformation of species data
No species-weights specified
No sample-weights specified
Centering/standardization by species = 1
Centering/standardization by samples = 0

No. of active samples: 23
No. of passive samples: 0
No. of active species: 137

Total sum of squares in species data = 366.587
Total standard deviation in species data TAU = 0.341086

****** Check on influence in covariable/environment data ******
The following sample(s) have extreme values

Sample	Environmental variable	Influence	Covariable influence	+ Environment space influence
18	3	42.6x		
18	6	5.2x		
18				3.2x
22	5	6.5x		

****** End of check ******

**** Start of forward selection of variables ****

*** Unrestricted permutation ***

Seeds: 23239 945

N	Name	Extra fit
12	Clay	0.04

6 Phosphate 0.08
5 Nitrate 0.09
11 Silt 0.12
3 SSS 0.13
2 SST 0.14
Environmental variable 2 tested
Number of permutations= 999

P-value 0.0040 (variable 2; F-ratio= 3.34; number of permutations= 999)

Environmental variable 2 added to model
Variance explained by the variables selected: 0.14
" " " all variables : 0.56

N Name Extra fit

12 Clay 0.05
6 Phosphate 0.10
11 Silt 0.12
5 Nitrate 0.13
3 SSS 0.25
Environmental variable 3 tested
Number of permutations= 999

P-value 0.0010 (variable 3; F-ratio= 8.01; number of permutations= 999)

Environmental variable 3 added to model
Variance explained by the variables selected: 0.38
" " " all variables : 0.56

N Name Extra fit

6 Phosphate 0.03
12 Clay 0.04
5 Nitrate 0.04
11 Silt 0.06
Environmental variable 11 tested
Number of permutations= 999

P-value 0.0170 (variable 11; F-ratio= 1.90; number of permutations= 999)

Environmental variable 11 added to model
Variance explained by the variables selected: 0.44
" " " all variables : 0.56

N Name Extra fit

6 Phosphate 0.03
12 Clay 0.04
5 Nitrate 0.04
Environmental variable 5 tested
Number of permutations= 999

P-value 0.0690 (variable 5; F-ratio= 1.54; number of permutations= 999)

Environmental variable 5 added to model
Variance explained by the variables selected: 0.48
" " " all variables : 0.56

N Name Extra fit

6 Phosphate 0.04
12 Clay 0.04
Environmental variable 12 tested
Number of permutations= 999

P-value 0.0940 (variable 12; F-ratio= 1.50; number of permutations= 999)

Environmental variable 12 added to model
Variance explained by the variables selected: 0.53
" " " all variables : 0.56

N Name Extra fit

6 Phosphate 0.04
Environmental variable 6 tested
Number of permutations= 999

P-value 0.1180 (variable 6; F-ratio= 1.39; number of permutations= 999)

Environmental variable 6 added to model
Variance explained by the variables selected: 0.56

"   "   "   all variables   :   0.56

No more variables to improve fit
*** End of selection ***

N	name	(weighted) mean	stand. dev.	inflation factor
1	SPEC AX1	0.0000	1.0256	
2	SPEC AX2	0.0000	1.0341	
3	SPEC AX3	0.0000	1.1250	
4	SPEC AX4	0.0000	1.2221	
5	ENVI AX1	0.0000	1.0000	
6	ENVI AX2	0.0000	1.0000	
7	ENVI AX3	0.0000	1.0000	
8	ENVI AX4	0.0000	1.0000	
2	SST	21.1497	1.3663	2.5470
3	SSS	33.1070	1.5214	4.3073
5	Nitrate	2.0589	0.8470	**5.5778**
6	Phosphate	0.2368	0.0477	4.3549
11	Silt	0.3901	0.1932	5.5411
12	Clay	0.1329	0.0624	4.9064

Note: "Nitrate" was removed from RDA model.

**** Summary ****

Axes	1	2	3	4
Total variance				
Eigenvalues :	0.316	0.100	0.063	0.038
1.000				
Species-environment correlations :	0.975	0.967	0.889	0.818
Cumulative percentage variance				
of species data :	31.6	41.6	47.9	51.7
of species-environment relation:	56.0	73.8	84.9	91.7

Sum of all eigenvalues
1.000
Sum of all canonical eigenvalues
0.564

[Wed Jul 08 13:55:29 2020] CANOCO call succeeded
[Wed Jul 08 13:55:47 2020] Settings changed

```
[Wed Jul 08 13:55:48 2020] Running CANOCO:
[Wed Jul 08 13:55:48 2020] CON file [D:\Canoco\spe.con] saved
 Program CANOCO Version 4.5 February 2002 - written by Cajo J.F. Ter Braak
 (C) 1988-2002 Biometris - quantitative methods in the life and earth sciences
 Plant Research International, Wageningen University and Research Centre
 Box 100, 6700 AC Wageningen, the Netherlands
 CANOCO performs (partial) (detrended) (canonical) correspondence analysis,
 principal components analysis and redundancy analysis.
 CANOCO is an extension of Cornell Ecology program DECORANA (Hill,1979)

 For explanation of the input/output see the manual or
 Ter Braak, C.J.F. (1995) Ordination. Chapter 5 in:
 Data Analysis in Community and Landscape Ecology
 (Jongman, R.H.G., Ter Braak, C.J.F. and Van Tongeren, O.F.R., Eds)
 Cambridge University Press, Cambridge, UK, 91-173 pp.

 *** Type of analysis ***
   Model                Gradient analysis
                  indirect      direct      hybrid
 linear          1=PCA        2= RDA        3
 unimodal         4= CA        5= CCA        6
    ,,            7=DCA        8=DCCA        9
                  10=non-standard analysis
 Type analysis number
 Answer =    2


 *** Data files ***
 Species data         : D:\Canoco\spe.dta
 Covariable data      :
 Environmental data : D:\Canoco\env.dta
 Initialization file:

 Forward selection of envi. variables =      1
 Scaling of ordination scores         =      2
 Diagnostics                          =      1

 File    : D:\Canoco\spe.dta
 Title   : WCanoImp produced data file
 Format :    (I5,1X,5F14.9,27(/6X,(5F14.9)))
 No. of couplets of species number and abundance per line :      0


 No samples omitted
```

Number of samples 23
Number of species 137
Number of occurrences 797

File : D:\Canoco\env.dta
Title : WCanoImp produced data file
Format : (I5,1X,4F15.9,3(/6X,(4F15.9)))
No. of environmental variables : 13

No interaction terms defined

Squareroot-transformation of species data
No species-weights specified
No sample-weights specified
Centering/standardization by species = 1
Centering/standardization by samples = 0

No. of active samples: 23
No. of passive samples: 0
No. of active species: 137

Total sum of squares in species data = 366.587
Total standard deviation in species data TAU = 0.341086

****** Check on influence in covariable/environment data ******
The following sample(s) have extreme values

Sample	Environmental variable	Influence	Covariable influence	+ Environment space influence
18	3	42.6x		
18	6	5.2x		
18				3.6x

****** End of check ******

**** Start of forward selection of variables ****

*** Unrestricted permutation ***

Seeds: 23239 945

N Name Extra fit

12 Clay 0.04
6 Phosphate 0.08
11 Silt 0.12
3 SSS 0.13
2 SST 0.14
Environmental variable 2 tested
Number of permutations= 999

P-value 0.0040 (variable 2; F-ratio= 3.34; number of permutations= 999)

Environmental variable 2 added to model
Variance explained by the variables selected: 0.14
" " " all variables : 0.52

N Name Extra fit

12 Clay 0.05
6 Phosphate 0.10
11 Silt 0.12
3 SSS 0.25
Environmental variable 3 tested
Number of permutations= 999

P-value 0.0010 (variable 3; F-ratio= 8.01; number of permutations= 999)

Environmental variable 3 added to model
Variance explained by the variables selected: 0.38
" " " all variables : 0.52

N Name Extra fit

6 Phosphate 0.03
12 Clay 0.04
11 Silt 0.06
Environmental variable 11 tested
Number of permutations= 999

P-value 0.0170 (variable 11; F-ratio= 1.90; number of permutations= 999)

Environmental variable 11 added to model
Variance explained by the variables selected: 0.44
" " " all variables : 0.52

N Name Extra fit

6 Phosphate 0.03
12 Clay 0.04
Environmental variable 12 tested
Number of permutations= 999

P-value 0.1320 (variable 12; F-ratio= 1.41; number of permutations= 999)

Environmental variable 12 added to model
Variance explained by the variables selected: 0.48
" " " all variables : 0.52

N Name Extra fit

6 Phosphate 0.03
Environmental variable 6 tested
Number of permutations= 999

P-value 0.2190 (variable 6; F-ratio= 1.21; number of permutations= 999)

Environmental variable 6 added to model
Variance explained by the variables selected: 0.52
" " " all variables : 0.52

No more variables to improve fit
*** End of selection ***

N	name	(weighted) mean	stand. dev.	inflation factor
1	SPEC AX1	0.0000	1.0357	
2	SPEC AX2	0.0000	1.0314	
3	SPEC AX3	0.0000	1.1896	
4	SPEC AX4	0.0000	1.1990	
5	ENVI AX1	0.0000	1.0000	

6	ENVI AX2	0.0000	1.0000	
7	ENVI AX3	0.0000	1.0000	
8	ENVI AX4	0.0000	1.0000	
2	SST	21.1497	1.3663	2.4296
3	SSS	33.1070	1.5214	3.7358
6	Phosphate	0.2368	0.0477	2.6941
11	Silt	0.3901	0.1932	**5.3635**
12	Clay	0.1329	0.0624	4.7633

Note: "Silt" was removed from RDA model.

**** Summary ****

Axes 1 2 3 4
Total variance

Eigenvalues : 0.309 0.100 0.046 0.036
1.000
Species-environment correlations : 0.965 0.970 0.841 0.834
Cumulative percentage variance
of species data : 30.9 40.9 45.5 49.1
of species-environment relation: 60.1 79.5 88.4 95.4

Sum of all eigenvalues
1.000
Sum of all canonical eigenvalues
0.515

[Wed Jul 08 13:55:50 2020] CANOCO call succeeded
[Wed Jul 08 13:56:14 2020] Settings changed
[Wed Jul 08 13:56:17 2020] Running CANOCO:
[Wed Jul 08 13:56:17 2020] CON file [D:\Canoco\spe.con] saved
Program CANOCO Version 4.5 February 2002 - written by Cajo J.F. Ter Braak
(C) 1988-2002 Biometris - quantitative methods in the life and earth sciences
Plant Research International, Wageningen University and Research Centre
Box 100, 6700 AC Wageningen, the Netherlands
CANOCO performs (partial) (detrended) (canonical) correspondence analysis,
principal components analysis and redundancy analysis.
CANOCO is an extension of Cornell Ecology program DECORANA (Hill,1979)

For explanation of the input/output see the manual or
Ter Braak, C.J.F. (1995) Ordination. Chapter 5 in:
Data Analysis in Community and Landscape Ecology
(Jongman, R.H.G., Ter Braak, C.J.F. and Van Tongeren, O.F.R., Eds)

Cambridge University Press, Cambridge, UK, 91-173 pp.

*** Type of analysis ***

Model	Gradient analysis		
	indirect	direct	hybrid
linear	1=PCA	2= RDA	3
unimodal	4= CA	5= CCA	6
,,	7=DCA	8=DCCA	9
	10=non-standard analysis		

Type analysis number
Answer = 2

*** Data files ***
Species data : D:\Canoco\spe.dta
Covariable data :
Environmental data : D:\Canoco\env.dta
Initialization file:

Forward selection of envi. variables = 1
Scaling of ordination scores = 2
Diagnostics = 1

File : D:\Canoco\spe.dta
Title : WCanoImp produced data file
Format : (I5,1X,5F14.9,27(/6X,(5F14.9)))
No. of couplets of species number and abundance per line : 0

No samples omitted
Number of samples 23
Number of species 137
Number of occurrences 797

File : D:\Canoco\env.dta
Title : WCanoImp produced data file
Format : (I5,1X,4F15.9,3(/6X,(4F15.9)))
No. of environmental variables : 13

No interaction terms defined

Squareroot-transformation of species data
No species-weights specified
No sample-weights specified
Centering/standardization by species = 1
Centering/standardization by samples = 0

No. of active samples: 23
No. of passive samples: 0
No. of active species: 137

Total sum of squares in species data = 366.587
Total standard deviation in species data TAU = 0.341086

****** Check on influence in covariable/environment data ******
The following sample(s) have extreme values

Sample	Environmental variable	Influence	Covariable influence	+ Environment space influence
18	3	42.6x		
18	6	5.2x		
18				4.3x

****** End of check ******

**** Start of forward selection of variables ****

*** Unrestricted permutation ***

Seeds: 23239 945

N	Name	Extra fit
12	Clay	0.04
6	Phosphate	0.08
3	SSS	0.13
2	SST	0.14

Environmental variable 2 tested
Number of permutations= 999

P-value 0.0040 (variable 2; F-ratio= 3.34; number of permutations= 999)

Environmental variable 2 added to model
Variance explained by the variables selected: 0.14
" " " all variables : 0.46

N Name Extra fit

12 Clay 0.05
6 Phosphate 0.10
3 SSS 0.25
Environmental variable 3 tested
Number of permutations= 999

P-value 0.0010 (variable 3; F-ratio= 8.01; number of permutations= 999)

Environmental variable 3 added to model
Variance explained by the variables selected: 0.38
" " " all variables : 0.46

N Name Extra fit

6 Phosphate 0.03
12 Clay 0.04
Environmental variable 12 tested
Number of permutations= 999

P-value 0.0860 (variable 12; F-ratio= 1.43; number of permutations= 999)

Environmental variable 12 added to model
Variance explained by the variables selected: 0.43
" " " all variables : 0.46

N Name Extra fit

6 Phosphate 0.03
Environmental variable 6 tested
Number of permutations= 999

P-value 0.3010 (variable 6; F-ratio= 1.16; number of permutations= 999)

Environmental variable 6 added to model
Variance explained by the variables selected: 0.46

"   "   "   all variables   :   0.46

No more variables to improve fit
*** End of selection ***

N	name	(weighted) mean	stand. dev.	inflation factor
1	SPEC AX1	0.0000	1.0473	
2	SPEC AX2	0.0000	1.0303	
3	SPEC AX3	0.0000	1.2257	
4	SPEC AX4	0.0000	1.2951	
5	ENVI AX1	0.0000	1.0000	
6	ENVI AX2	0.0000	1.0000	
7	ENVI AX3	0.0000	1.0000	
8	ENVI AX4	0.0000	1.0000	
2	SST	21.1497	1.3663	1.8592
3	SSS	33.1070	1.5214	2.7648
6	Phosphate	0.2368	0.0477	2.6933
12	Clay	0.1329	0.0624	1.0194

Note: The values of VIF varied from 1~3, indicating the low collinearity between the environmental variables.

**** Summary ****

**Axes**	**1**	**2**	**3**	**4**	**Total variance**
**Eigenvalues:**	**0.300**	**0.099**	**0.039**	**0.024**	**1.000**
**Species-environment correlations:**	**0.955**	**0.971**	**0.816**	**0.772**	
**Cumulative percentage variance**					
**of species data:**	**30.0**	**39.9**	**43.8**	**46.2**	
**of species-environment relation:**	**65.1**	**86.5**	**94.8**	**100.0**	
**Sum of all eigenvalues**					**1.000**
**Sum of all canonical eigenvalues**					**0.462**

[Wed Jul 08 13:56:19 2020] CANOCO call succeeded
